# Starch-Rich Diet Induced Rumen Acidosis and Hindgut Dysbiosis in Dairy Cows of Different Lactations

**DOI:** 10.3390/ani10101727

**Published:** 2020-09-23

**Authors:** Viktoria Neubauer, Renee M. Petri, Elke Humer, Iris Kröger, Nicole Reisinger, Walter Baumgartner, Martin Wagner, Qendrim Zebeli

**Affiliations:** 1 Unit of Food Microbiology, Institute of Food Safety, Food Technology, and Veterinary Public Health, University of Veterinary Medicine, 1210 Vienna, Austria; Martin.wagner@vetmeduni.ac.at; 2 FFoQSI GmbH—Austrian Competence Centre for Feed and Food Quality, Safety & Innovation, 3430 Tulln, Austria; 3 Institute of Animal Nutrition and Functional Plant Compounds, University of Veterinary Medicine, 1210 Vienna, Austria; renee.petri@canada.ca (R.M.P.); elke.humer@donau-uni.ac.at (E.H.); iris_kroeger@gmx.de (I.K.); Qendrim.Zebeli@vetmeduni.ac.at (Q.Z.); 4 Department for Psychotherapy and Biopsychosocial Health, Danube University Krems, 3500 Krems, Austria; 5 BIOMIN Research Center, BIOMIN Holding GmbH, 3430 Tulln, Austria; nicole.reisinger@biomin.net; 6 University Clinic for Ruminants, University of Veterinary Medicine, 1210 Vienna, Austria; Walter.Baumgartner@vetmeduni.ac.at

**Keywords:** fecal-microbiome, fecal-pH, hindgut-acidosis, subacute-rumen-acidosis, short-chain-fatty-acids, parity, high-concentrate

## Abstract

**Simple Summary:**

High-producing dairy cows receive high-energy diets for maintenance and production. This study showed that 60% concentrate in the diet, containing 27.7% starch, changed the fecal-microbial community and lowered its diversity, suggesting hindgut dysbiosis. Both ruminal and fecal pH decreased with high-starch feeding, which suggests further investigations in fecal pH as rumen- and hindgut-acidosis diagnostic tool. Cows in the third lactation spent more time below the threshold for subacute-ruminal acidosis (pH 6.0) than second or fourth-or-below lactation cows. Their higher susceptibility was caused by their high dry matter intake but missing counter-regulation by increased rumination activity. Further, we suggest that body weight and rumen size might play a role in the absorptive capacity of short-chain fatty acids. The study also identified indicator-bacterial phylotypes that changed with starch-rich diet and lactation number. In conclusion, we suggest including lactation number as a factor in practical feeding management for identification of high risk-cows for acidosis, and in dairy cow research.

**Abstract:**

Starch-rich diets can cause subacute ruminal acidosis (SARA) in dairy cows with potentially different susceptibility according to lactation number. We wanted to evaluate the bacterial community and the fermentation end products in feces to study susceptibility to hindgut acidosis and dysbiosis. Sixteen dairy cows received a medium-concentrate diet (MC, 40% concentrate, 18.8% starch) for one week and a high-concentrate diet (HC, 60% concentrate, 27.7% starch, DM) for four weeks. Milk yield, dry-matter intake, chewing activity, ruminal pH, milk constituents, and fecal samples for short-chain fatty acids (SCFA), pH, and 16S rRNA-gene sequencing were investigated. The HC feeding caused a reduction in fecal pH, bacterial diversity and richness, an increase in total SCFA, and a separate phylogenetic clustering of MC and HC samples. Ruminal and fecal pH had fair correlation (*r* = 0.5). Cows in the second lactation (2ndL) had lower dry matter intake (DMI) than cows of third or fourth or more lactations (3rdL; ≥4 L), whereas DMI/kg body weight was lower for ≥4 L than for 2ndL and 3rdL cows. The mean ruminal pH was highest in ≥4 L, whereas the time spent below the SARA threshold was highest for 3rdL cows. The latter also had higher total SCFA in the feces. Our results suggest that hindgut dysbiosis is caused by increased substrate flow to the hindgut, but further investigations are needed to define hindgut acidosis. The 3rdL cows were most susceptible to rumen acidosis and hindgut dysbiosis due to high DMI level, but missing counter regulations, as suggested happening in 2ndL and ≥4 L cows.

## 1. Introduction

High-producing dairy-cow diets typically consist of large amounts of starch-containing concentrates to meet their demands for glycogenic precursors such as propionate [1]. However, excessive production of short-chain fatty acids (SCFA) decreases ruminal pH and can lead to subacute-ruminal acidosis (SARA), with an accompanied dysbiosis and increased absorption rate at the rumen epithelium [2]. The syndrome can include impaired cow health, including local and systemic inflammation, impaired liver health, abscess formation, laminitis, and reduced production levels [3,4,5]. 

Disturbed rumen conditions are associated with higher osmolarity into the lumen and higher passage rate, which leads to greater amounts of undigested substrates passing to the hindgut (caecum, colon, and rectum) [6]. In general, the reticulorumen and the hindgut in cattle do share a variety of physiological similarities and react similarly to dietary perturbations. Increased by-pass starch leads to increased fermentation activity in the hindgut as well, with similar effects as in the rumen, including bacterial community shifts, increased SCFA production, and a decrease in pH [6]. As in the rumen, the absorption rate of SCFA also increases with decreasing pH in the hindgut. However, the hindgut lacks saliva and there is less bicarbonate exchange when luminal pH drops in the hindgut. Thus, buffering capacity in the hindgut is more limited than in the rumen [6]. The protective mucus layer has a narrow pH tolerance of around 7.0 and is more susceptible to luminal dysbiotic conditions than the keratinized epithelial cells in the rumen [6]. Acidic conditions in the hindgut have an impact on mucosal permeability and integrity [7], and therefore contribute significantly to systemic health issues [6,8]. Most of the prior research has focused on rumen acidosis and less information is available about hindgut dysbiosis caused by bypass carbohydrates during starch-rich feeding. The fecal analysis gives the best opportunity to study hindgut dysbiosis as it is both representatives for the hindgut and a non-invasive method of examination [9]. Despite the ease of analysis of feces, there are very few studies describing the correlation of ruminal and fecal pH, and the associated changes in the fecal-microbial community and its fermentation products with high-concentrate diets.

Research in recent years has indicated different susceptibilities to SARA among cows in different lactations [10,11]. One contributing factor to lesser susceptibility is an increase of the rumen-mucosa thickness, and thereby absorptive capacity, with the duration of concentrate feeding and with increased lactation numbers [12,13]. Another major contributing factor is the adaptation of the gut microbiome, of which the rumen and fecal microbiome vary in high and low producing dairy cattle [14,15] and with age [16]. 

The first aim of this study was to induce a reticuloruminal-pH drop in the cows and describe changes of pH, SCFA, and microbial community in the feces, to get a better understanding of hindgut acidosis and an associated dysbiosis. The second aim was to determine how the number of lactations of the cows correlates to the susceptibility of rumen and hindgut acidosis and associated dysbiosis. We hypothesized that the fecal pH, SCFA, and microbial community will be impaired due to the inclusion of 60% concentrate in the diet, signifying a hindgut acidosis and dysbiosis. Our second hypothesis was that cows with a higher number of lactations are less susceptible to high-concentrate inclusion and their fecal microbiota show fewer changes due to dietary challenges.

## 2. Materials and Methods

All procedures involving animal handling and treatment were approved by the institutional ethics committee of the University of Veterinary Medicine Vienna and the national authority according to section 26 of the law for animal experiments 2012-TVG (GZ: BMWFW-68.205/0098- WF/V/3b/2016).

### 2.1. Experimental Setup, Animals, and Feeding

Sixteen lactating Simmental cows (739 ± 84.6 kg BW; 35.6 ± 4.7 kg milk per day; 3.5 ± 1.66 lactations, 86 ± 20.7 days in milk (DIM) ± SD), housed in a free-stall barn with straw bedding at the research and teaching farm of the Vetmeduni Vienna (Vetfarm, Pottenstein, Austria), were used for this experiment. They were first fed a medium-concentrate diet with 40% concentrate and 60% forage, resulting in 18.8% starch, on DM basis for 1 week (day −6–0; MC). Then they were switched abruptly to a high-concentrate diet with 60% concentrate, 40% forage, resulting in 27.7% starch, on DM basis, fed for four weeks (day 1–28; HCwk1–4). Cows were enrolled to the study in two consecutive experimental blocks (n = 6, n = 10). The forage diet was based on grass-alfalfa silage and meadow-hay at a ratio of 80:20 on DM basis. The concentrate was based on barley (63%), soybean meal (15%), corn (9%), solvent-extracted canola-meal (8%), and mineral-vitamin supplements. Exact feed composition and chemical analysis of the diets is published in [17]. The diet was offered twice daily (07:30, 17:30) as a total mixed ration for ad libitum intake in automated feeding troughs (Insentec B.V., Marknesse, the Netherlands), that continuously measured feed intake. Cows had free access to fresh water and salt lick stones. The cows were milked twice daily (07:00, 17:00) and milk yield was recorded automatically (Alpro Milking, DeLaval Inc., Kansas City, MO, USA). Milk-content samples were taken once per week and analyzed for fat, protein, urea, somatic-cell count, lactose, and pH (Combifoss (Foss, Hillerød, Denmark). Reticular pH was measured continuously with indwelling rumen-pH boli (Smaxtec Animal Care GmbH, Graz, Austria). Rumination activity was measured during the last four days in MC (d −3–0), HCwk1 (d 1–6), and HCwk4 (d 25–27), using noseband-sensor halters (RumiWatch System, ITIN+Hoch GmbH, Liestal, Switzerland). The data was processed using RumiWatch Manager 2 (V2.1.0.0.; RumiWatch System, ITIN+Hoch GmbH, Liestal, Switzerland) as described by [17]. A clinical examination of all cows was carried out at the beginning of the experiment by the performing veterinarians. This resulted in no clinical findings in the beginning, nor showed any of the cows’ clinical signs during the experiment. This study is part of a larger research project. Therefore, results of performance, milk constituents, ruminal pH, dry matter intake (DMI), chewing activity, and sorting along the feeding model are described in [17].

### 2.2. Fecal Sampling and Fermentation Parameters

Fecal samples of all cows were taken at the end of MC feeding (d0), and during HCwk2, wk3, and wk4 (d12, d20, d28) before morning milking. Feces were sampled rectally using a clean rectal glove for each animal. Subsamples were put into 2 mL Cryo-tubes for microbial-DNA-analysis and into 8 mL tubes (Sarstedt AG, Wiener Neudorf, Austria) for SCFA and pH analysis, using disinfected tweezers. Samples for DNA extraction were frozen at −80 °C, for SCFA and pH analysis at −20 °C until further processing.

For fecal pH measurement, 1.0 g of thawed feces was diluted with 9.0 mL distilled water and pH was measured in triplicate using a hand-held pH meter (SevenGoMultiTM, Mettler Toledo, Vienna, Austria).

Concentrations of SCFA (acetate, propionate, butyrate, valerate, caproate, iso-butyrate, iso-valerate) were determined by GC (GC Model 8060 MS172DPFC, no. 950713, Fisons, Rodena, Italy) as previously described [18]. The fecal samples were thawed, centrifuged 25 min at 20,000× *g* at 4 °C, the supernatant transferred into a fresh tube with 0.2 mL HCl (1.8 mol/L), the internal standard (0.2 mL; 4-methylvaleric acid, Sigma-Aldrich Co. LLC, St. Louis, MO, USA) added, centrifuged again (25 min, 20,000× *g*, 4 °C), and the clear supernatant transferred into the GC vial. A flame-ionization detector and a 30 m × 0.53 mm ID × 0.53 μm df capillary column (Trace TRWax, Thermo Fisher Scientific, Waltham, MA, USA) were used. As reference, an external standard for each targeted acid with a known concentration was used. Generation and evaluation of chromatograms was done with Stratos Software (Stratos Version 4.5.0.0, Polymer Laboratories, Church Stretton, Shropshire, UK).

### 2.3. DNA Extraction and Sequencing

For DNA extraction and sequencing, only samples from MC, HCwk2, and HCwk4 were investigated. A 210 mg fecal sample was processed with the QIAamp Fast DNA Stool Mini Kit (Qiagen, Hilden, Germany), using the protocol for isolation of DNA from stool for pathogen detection according to the manufacturer’s instructions, with 95 °C at the heating step. Total DNA quantity after isolation was measured for all samples using a Qubit Fluorometer 2.0 (Qubit dsDNA HS Assay Kit, Thermo Fisher Scientific, Vienna, Austria) according to the manufacturer’s instructions, resulting in 6.3 ng/µL on average per sample. 

Illumina MiSeq paired-end amplicon sequencing (Microsynth AG, Balgach, Switzerland) was conducted to target the bacterial 16S rRNA gene hypervariable regions V3–5. The primer set 357F (5′-CCTACGGGAGGCAGCAG-3′) and 926R (5′-CCGTCAATTCMTTTRAGT-3′ [19] was used to generate an approximate amplicon size of 570 bp. Multiplexed libraries were generated by ligating sequencing adapters and indexes onto purified PCR products (Nextera XT Sample Preparation Kit, Illumina, Inc., San Diego, CA, USA)). After sequencing, primers were trimmed and corresponding overlapping paired-end reads were stitched by Microsynth (Microsynth AG, Balgach, Switzerland). 

The sequencing data were deposited into the European Nucleotide Archive (ENA) and can be accessed via the study accession number PRJEB39473 [20].

### 2.4. Bioinformatic Analysis

Sequence data were analyzed using QIIME (version 1.9.1. http://qiime.org/) [21], based on the recommended workflows of QIIME tutorials [22] and the authors of [23]. After a first downstream analysis, two samples (both HCwk2, one 2ndL, one 3rdL cow) were removed completely from the dataset due to a goods coverage below 90% [24]. The remaining dataset (4,249,393 sequences) was re-analyzed. Sequences were trimmed with a quality score of 20 (q19) using the command split libraries. The chimeric sequences were identified using check chimeras, the gold.fa reference database [25], and *usearch* (version 11.0) [26], and subsequently filtered. A total of 998,630 sequences passed quality control and chimera check. Sequences were then clustered into operational taxonomic units (OTU) with a 97% 16S rRNA gene similarity cutoff and a minimum sequence number of 10. This was performed by open-reference OTU picking against SILVA database [27] and *usearch*. A total of 4746 OTU were assigned, 99 OTU were removed due to missing taxonomic information on kingdom level. The remaining 4647 OTU, with a total number of 859,749 sequences (20% of raw sequence dataset), a mean number of 18,690 ± 5036 sequences per sample, were used for further taxonomic-based analyses on phylum and family level. Measures of α-diversity were determined using the alpha rarefaction command in QIIME, including species richness estimator Chao1, diversity indices Shannon and Simpson, singles, and goods coverage. The rarefaction depth was equalized for all samples to 9903 sequences per sample based on the minimum sequence number achieved. For β-diversity analysis, the weighted UniFrac distance matrix was calculated with the beta diversity command, also normalized for 9903 sequences, and issued as 3D principal coordinate analysis (PCoA) plots.

### 2.5. Statistical Analysis

Statistical analyses for DMI, milk yield, milk composition, chewing activity, ruminal pH, fecal pH, fecal SCFA, phyla, families, and alpha-diversity-parameters were performed with MIXED PROC in SAS software (version 9.4; SAS Institute Inc., Cary, NC, USA) using a variance-components model. Cows were grouped according to their number of lactation (second lactation, 2ndL, *n* = 5; third lactation, 3rdL, *n* = 6; four or more lactations, ≥4 L, *n* = 5, mean = 5.6 lactations). Feeding phase (MC, HCwk1–4) and lactation group were considered as fixed effects, cow within a phase as a repeated effect, experimental block as a random effect. Post hoc test was performed using the pdiff option, *p*-values of >0.05–<0.1 are considered as a trend, *p* ≤ 0.05 as significant. Correlation analysis was performed using PROC CORR in SAS Enterprise Guide software (version 7.11; SAS Institute Inc., Cary, NC, USA), calculating Spearman rank correlation r. The r is considered as poor with |<0.3|, fair with |≥0.3–<0.5|, moderate with |≥0.5–<0.7|, strong with |≥0.7–<0.9|, and substantial with |≥0.9–1.0|. All correlation given are significant with *p* < 0.01 [28]. Figures and Tables were constructed using Excel (Excel 2016) and QIIME (version 1.9.1.). 

## 3. Results

### 3.1. Effects of Feeding an Increased Starch Level on Fecal pH and SCFA

**Figure 1:**
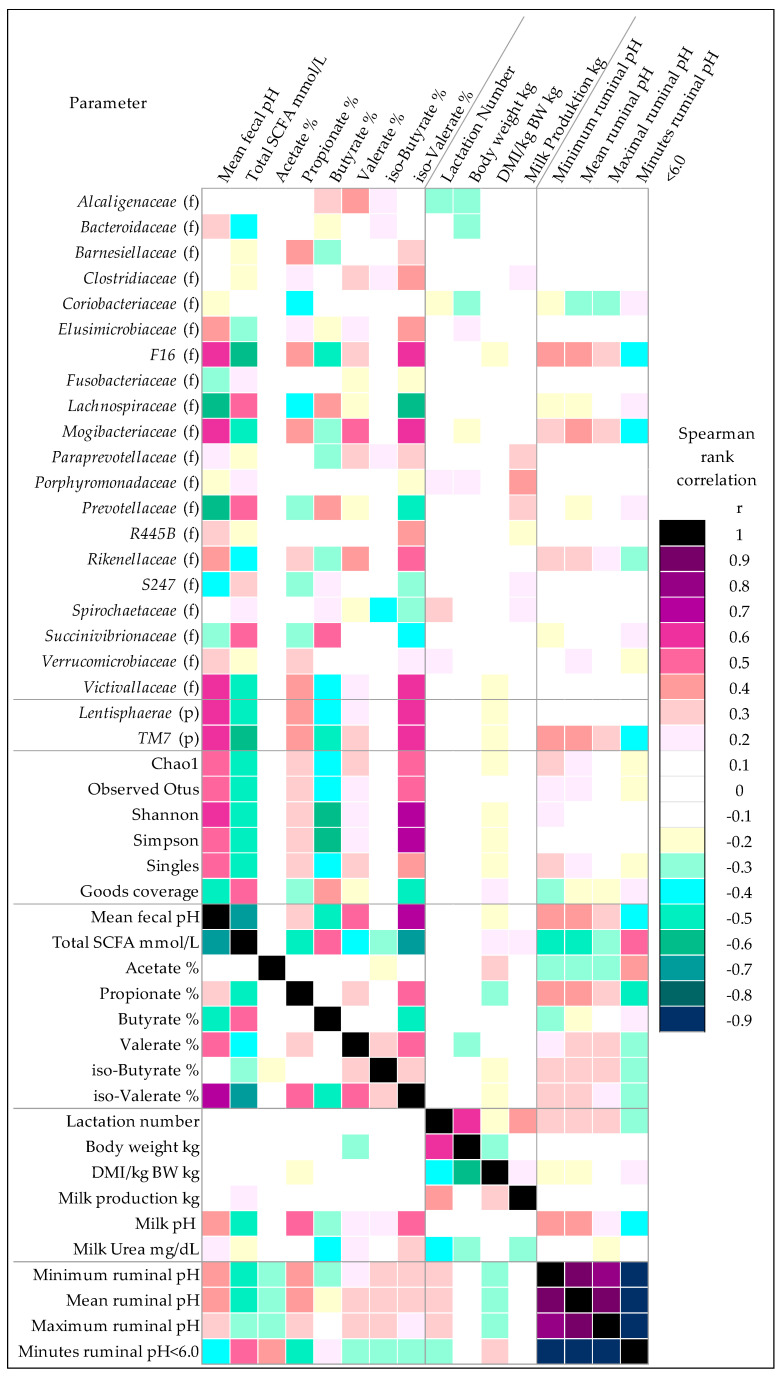
Spearman rank correlation (r) analysis between bacterial families, bacterial phyla, alpha-diversity-parameters, fecal pH, fecal SCFA, performance parameters, milk composition, and ruminal pH. Only interactions that had at least one r > 0.5 and *p* < 0.01 are given in the figure. (f) bacterial family; (p) bacterial phylum.

The feeding of HC diet increased total SCFA in the feces and decreased the fecal pH, with these two parameters having a strong negative correlation (*r* = −0.72). The SCFA profile changed with HC feeding towards less propionate, valerate, and iso-valerate, and more butyrate and caproate in the feces. Acetate showed only a decreasing trend in the HCwk2 (Table 1). Accordingly, propionate (*r* = −0.50), valerate (*r* = −0.42), and iso-valerate (*r* = −0.72) correlated negatively with total SCFA, and valerate (*r* = 0.51) and iso-valerate (*r* = 0.76) positively with fecal pH. Butyrate correlated positively with total SCFA (*r* = 0.55), and negatively with fecal pH (*r* = −0.51), whereas acetate, caproate, and iso-butyrate had no relevant correlations (Figure 1). Fecal pH showed a fair positive correlation with mean and minimum ruminal pH (*r* = 0.49 and 0.45, respectively) and a fair negative one with the time cows spent below a ruminal pH of 6.0 (*r* = −0.47; Figure 1). There was no significant correlation between fecal pH and DMI, milk production, or milk contents. Only milk pH showed fair to high correlations with fecal pH (*r* = 0.43), ruminal pH (*r* = 0.46), and different SCFA (Figure 1).

### 3.2. Effects of Feeding an Increased Starch Level on the Fecal Microbiome

**Figure 2:**
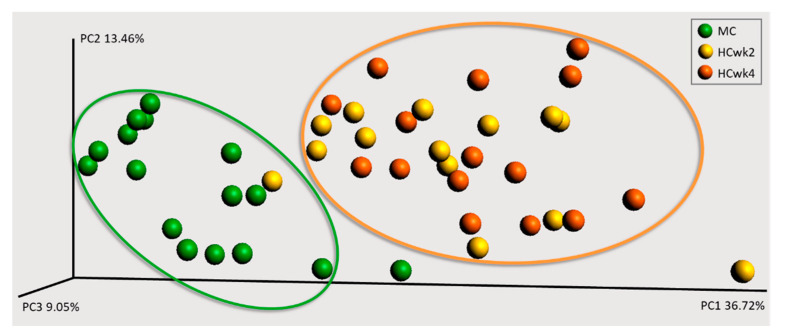
Three-dimensional principal coordinate analysis (PCoA) plots based on weighted UniFrac distance metric, showing the phylogenetic clustering of fecal 16S rRNA gene amplicon sequencing. 16 dairy cows were fed a moderate-concentrate diet (MC, 40% concentrate, 18.8% starch, DM, for 1wk), followed by 4wks of high-concentrate feeding (HCwk 2&4, 60% concentrate, 27.7% starch, DM). The three axes [principal component (PC) 1, 2, 3] indicate the variation between the samples in percent and are scaled according to their percentage explained.

Alpha diversity parameters Shannon, Simpson, Chao1, and the number of singletons decreased with HC feeding, and showed a strong positive correlation with fecal pH and iso-valerate, and strong negative correlation with total SCFA and butyrate. The goods coverage increased with HC feeding and correlated strongly inversely with those parameters (Figure 1, Table 2). Calculation of the weighted UniFrac distance metric revealed a separate clustering of MC samples, whereas HCwk2 and HCwk4 clustered together (Figure 2). 

**Figure 3:**
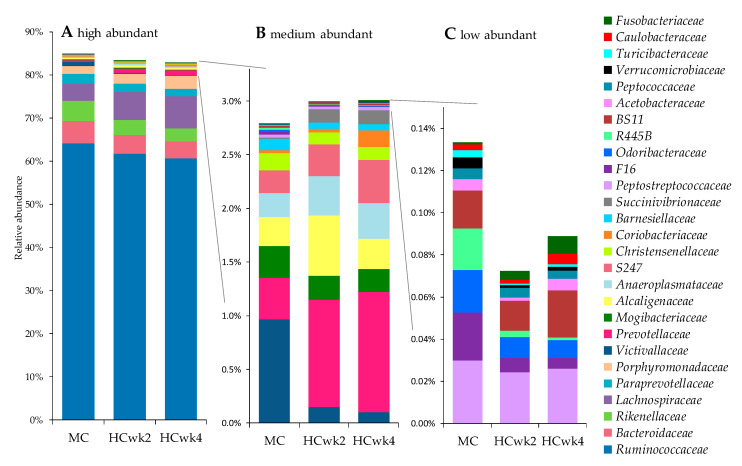
Bacterial families that changed in relative abundance along the feeding model. *Caulobacteriaceae*, RF16, and *Spirochaetaceae* changed by trend (0.05 < *p* < 0.1), all others significantly (*p* ≤ 0.05). MC moderate concentrate diet (40% concentrate, 18.8% starch, fed for one wk), HCwk1–4 high concentrate diet (60% concentrate, 27.7% starch, fed for four wk); (**A**) all families, (**B**) medium abundant families, (**C**) low abundant families extracted.

The three highest abundant phyla found in the fecal samples were *Firmicutes* (75.1% total relative abundance), *Bacteroidetes* (21.8%), and *Proteobacteria* (1.1%). The other Phyla were below one percent total relative abundance in the whole dataset (*Lentisphaerae* 0.39%, *Spirochaetes* 0.48%, *Tenericutes* 0.55%, *Cyanobacteria* 0.32%, *Fibrobacteres* 0.21%, *Actinobacteria* 0.08%, *Elusimicrobia* 0.02%, *TM7* 0.01%, *Fusobacteria* 0.004%, *Verrucomicrobia* 0.003%). From the three highest abundant phyla, *Proteobacteria* was influenced by the feeding model by decreasing from HCwk2 to HCwk4 by trend (−1.34-fold, *p* = 0.06). Among the low abundant phyla, *Lentisphaerae* decreased from MC to HCwk2 by −5.98-fold and from MC to HCwk4 by −9.82-fold (*p* < 0.01), with moderate correlation to fecal pH (*r* = 0.62) and total SCFA (*r* = −0.54). *TM7* decreased from MC to HCwk2 by −3.14-fold and from MC to HCwk4 by −3.88-fold (*p* < 0.01) with moderate correlation to fecal pH (*r* = 0.69) and SCFA (*r* = −0.61) and fair correlation with ruminal pH (*r* = 0.42; Figure 1). *Verrucomicrobia* decreased from MC to HCwk2 by −4.25-fold, from MC to HCwk4 by −3.00-fold (*p* < 0.01) with fair correlation with fecal pH (*r* = 0.32). *Actinobacteria* increased from MC to HCwk4 by 5.47-fold and from HCwk2 to HCwk4 by 4.74-fold (*p* < 0.01) with fair correlation to ruminal pH (*r* = −0.35) and propionate (*r* = −4.3). *Tenericutes* increased from MC to HCwk2 by trend (1.3-fold, *p* = 0.05), and *Fusobacteria* increased from MC to HCwk4 by 8.33-fold (*p* < 0.01) and from HCwk2 to HCwk4 by trend (2.02-fold, *p* = 0.07). From all 4647 OTU found, 88.6% could be assigned to 38 different families, with *Ruminococcaceae* (61.9%), *Lachnospiraceae* (6.2%), and *Bacteroidacaeae* (4.5%) being the three highest abundant families assigned (Supplemental Figure S1). Among all assigned families, 24 changed significantly and three by trend in relative abundance with the HC feeding (Figure 3). From the families influenced by the feeding model, *Lachnospiraceae* and *Prevotellaceae* had a strong negative correlation with fecal pH and iso-valerate, and a strong positive correlation with total SCFA. *Succinivibrionaceae* showed a strong positive correlation with total SCFA and butyrate, and a fair negative correlation with fecal pH and propionate (Figure 1). Family F16, *Victivallaceae*, and *Mogibacteriaceae* had a strong positive correlation with fecal pH and iso-valerate, a fair positive correlation with propionate, a strong negative correlation with total SCFA, and a strong to fair negative correlation with butyrate. *Rikenellaceae*, R445B, *Bacteroidaceae*, and *Verrucomicrobiaceae* had fair correlations with fecal pH, total SCFA, and partly iso-valerate (Figure 1).

### 3.3. Effects of Lactation Number on Performance, Ruminal pH, Fecal pH, and SCFA

Over the course of the whole experiment, the 3rdL and ≥4 L cows produced on average 4.75 kg more milk and consumed 2.72 kg more DM per day than 2ndL cows (*p* < 0.01). The number of lactations had a fair correlation with milk kg (*r* = 0.49). The BW increased with increasing lactation group (650, 757, 809 kg ± 29.5 kg SEM in 2ndL, 3rdL, ≥4 L cows, respectively, *p* < 0.01) and correlated moderately with number of lactations (*r* = 0.60). The DMI/kg BW was significantly lower in ≥4 L cows (3.02 kg) than in 2ndL and 3rdL cows (3.38, 3.29 kg respectively, averaged over the whole experiment, *p* = 0.01), with a fair correlation between the number of lactations and DMI/kg BW (*r* = −0.45). The milk yield per kg DMI did not differ between lactation groups. The 3rdL cows were 80 DIM at the start of the experiment, which was significantly less than 2ndL (91 DIM) and ≥4 L cows (88 DIM; SD ± 20.5 days, SEM ± 1.5; *p* < 0.01). Both milk-urea nitrogen and milk-urea nitrogen per kg DMI were lower in ≥4 L cows (19.5 mg/dL; 0.8 mg/kg DMI) than in 2ndL and 3rdL cows (23.3, 22.1 mg/dL; 1.1, 0.9 mg/kg DMI, respectively; *p* < 0.01) on average along the whole experiment. Milk urea nitrogen showed a fair correlation with lactation number and BW (*r* = −0.44, −0.30 respectively). The ≥4 L cows had on average higher somatic-cell count than 2ndL and 3rdL, and lower lactose than 2ndL cows (Table 3). 

On average, over the course of the whole experiment, ≥4 L cows spent fewer minutes eating, total chewing, and had less eating-chews/kg DMI than 2ndL and 3rdL, and less eating time/kg DMI than 2ndL cows (*p* < 0.05; Table 3, Table S1). The 2ndL cows had less rumination boli, but more chews per bolus than 3rdL cows (*p* < 0.2; Supplemental Table S1). The 2ndL cows had more total chewing time/kg DMI, ruminating chews/g DMI, eating chews/g DMI and total chews/g DMI than 3rdL and ≥4 L cows (*p* < 0.02; Supplemental Table S1). The 2ndL cows had higher rumination time/kg DMI than 3rdL cows, and ≥4 L cows had intermediate values with no significant difference to the other lactation groups (Table 3). On average over the whole experiment, the mean and minimum ruminal pH were higher in ≥4 L cows (6.53; 6.09, respectively) compared to 2ndL (6.28; 5.81) and 3rdL cows (6.20; 5.72; *p* < 0.01). The maximum pH and the minutes below pH 6.0 differed between the three lactation groups, in the order ≥4 L (max pH 7.00, 99 min pH < 6.0), 2ndL (max pH 6.68, 374 min pH < 6.0) and 3rdL (max pH 6.49, 606 min pH < 6.0; *p* < 0.01). All rumen-pH values had a fair correlation to the number of lactations. There was no effect of lactation group on fecal pH (*p* = 0.8), but total SCFA were higher in 3rdL (84.6 mmol/L) than in ≥4 L cows (72.2 mmol/L; *p* = 0.02), and valerate higher in 2ndL (1.16%) than 3rdL (1.0%) and ≥4 L cows (1.03%; *p* = 0.04) on average over the course of the whole experiment. Furthermore, acetate, propionate, caproate, iso-butyrate, and iso-valerate showed lactation-group effects in different feeding phases (Table 3).

### 3.4. Effects of Lactation Number on the Fecal Microbiome

There was no significant effect of lactation group on alpha diversity parameters, nor a clear clustering among the PCoA plots. Goods coverage reached >90% for the lactation groups in all feeding phases, which indicates a reliable sampling depth to analyze for fixed effects. The phylum *Bacteroidetes* was higher in ≥4 L than 2ndL cows (HCwk4, 1.27-fold, *p* = 0.03), *Elusimicrobia* higher in ≥4 L than 3rdL cows (HCwk4, 6.31-fold, *p* = 0.04), and *Spirochaetes* higher in ≥4 L cows than 2ndL (overall 1.46-fold, *p* = 0.02; in HCwk2 1.64-fold, *p* = 0.06) and 3rdL (MC, 2.01-fold, *p* = 0.02; respectively). The phylum *Lentisphaerae* was higher in 3rdL than in ≥4 L cows during MC (2.09-fold, *p* < 0.01). *Firmicutes*, *Actinobacteria*, and *Proteobacteria* were higher in 2ndL than ≥4 L cows in HCwk4 by trend (1.06, 2.33, 1.80-fold, respectively, *p* = 0.08). *Tenericutes* were higher in 2ndL than 3rdL cows by trend (overall 1.25-fold, *p* = 0.08, HCwk2 1.44-fold, *p* = 0.09).

**Figure 4:**
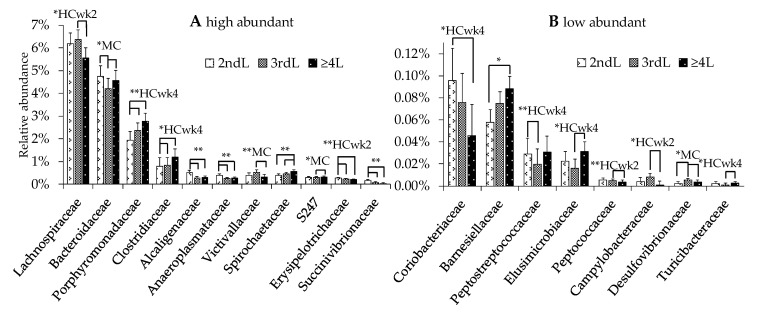
High (**A**) and low (**B**) abundant family distribution in feces of cows in the second (2ndL), third (3rdL), or fourth or higher lactation (≥4 L). The data includes all three feeding phases, superscripts indicate significant difference (** *p* ≤ 0.05) or trend (* 0.05 > *p* < 0.01) and the feeding phase where the statistical effect occurred (MC, moderate, 40% concentrate diet, 18.8% starch, DM), HCwk2, HCwk4 (High, 60% concentrate, 27.7% starch, DM, week 2 and 4); when no feeding phase is given, the effect occurred along the whole feeding experiment.

Ten families were influenced by lactation group by trend (0.05 > *p* < 0.1) and nine significantly (*p* ≤ 0.5; Figure 4). Amongst significant effects, the 2ndL cows had higher *Alcaligenaceae* (1.93, 1.78-fold; *r* = −0.33 with BW and lactation number), *Anaeroplasmataceae* (1.55, 1.48-fold), *Erysipelotrichaceae* (HCwk2, 1.56, 1.60-fold), and *Succinivibrionaceae* (HCwk4, 2.85, 11.77-fold), and lower *Porphyromonadaceae* (HCwk4, 2.00, 2.01-fold) than 3rdL or ≥4 L cows, respectively. *Peptostreptococcaceae* were higher in 2ndL than in 3rdL cows (HCwk4, 10.18-fold). The ≥4 L cows had higher *Spirochaetaceae* than 2ndL and 3rdL cows (1.47, 1.23-fold respectively; *r* = 0.32 with lactation number), and lower *Victivallaceae* (MC, 2.08-fold) and *Peptococcaceae* (HCwk2, 100% less) than 3rdL cows. Amongst trend effects, 2ndL cows had higher *Coriobacteriaceae* (HCwk4, 2.33-fold; *r* = −0.32 with BW) and lower *Barnesiellaceae* (1.53-fold) than ≥4 L cows, and lower *Clostridiaceae* than 3rdL and ≥4 L cows (HCwk4, 1.04, 2.90-fold respectively). The 3rdL cows had higher *Desulfovibrionaceae* than 2ndL and ≥4 L (MC, 3.61, 3.50-fold), higher *Lachnospiraceae* (HCwk2, 1.34-fold), and *Campylobacteraceae* (HCwk2, 16-fold) than ≥4 L, and lower *Bacteroidaceae* (MC, 1.23, 125-fold; *r* = −0.37 with BW) than 2ndL and ≥4 L, lower S247 (MC, 2.09-fold), *Elusimicrobiaceae* (HCwk4, 5.61-fold), and lower *Turicibacteraceae* (HCwk4, 100% less) than ≥4 L cows.

## 4. Discussion

This study aimed to investigate the effect of high-starch feeding on fecal pH and microbiome, and so describe the dysbiosis associated with hindgut acidosis in dairy cows. Special emphasis was put on susceptibility to rumen acidosis and hindgut dysbiosis in relation to the number of lactations.

### 4.1. High-Concentrate Feeding Causes Hindgut Dysbiosis

The change from a moderate concentrate (40% DM) diet to a high-concentrate (60%, DM) diet caused on average a decrease in reticular pH below the threshold of 6.0 as an indicator for SARA, as published in our accompanying paper [17]. The prolonged feeding of HC probably caused an increased flow of carbohydrates to the hindgut and resulted in the fecal pH decrease by 0.4 down to 7.4 in HCwk4. This goes along with other studies that showed a decrease of fecal pH by 0.7 to 6.42 with the increase from 40 to 70% concentrate in the diet [29], or by 0.2 to a pH of 6.45 with a change from 30% to 64% grain-based diet [3]. Currently, there is no fecal-pH threshold defined for hindgut acidosis, nor an indicator fecal pH for rumen acidosis. However, the relevant correlation of approximately 0.5 between ruminal-pH values and fecal-pH values defines dependency. Continuous measurements of ruminal pH and rumination activity clearly show that these parameters are subjected to diurnal variations, depending on feeding time points [17]. Only a few studies investigated diurnal changes in fecal pH. Sulzberger et al. [30] showed that ruminal pH was lowest 8 h after a grain challenge, and fecal pH 16 h after (decreased from approximately 6.4 to 5.5), and the mean difference was 0.1 pH values. However, it remains unclear, if the fecal pH also recovers over-night, like the ruminal pH does, and how this is impacted by passage rate and retention time. To define a threshold for hindgut acidosis—including a revised difference between ruminal and fecal pH, and an optimal time-point for fecal sampling—continuous measurements will be necessary.

The general proportion of the SCFA fractions was similar to what we found in rumen-digesta samples [31]. Although clear shifts of the bacterial community were seen between MC and HC diets, and total SCFA increased, the SCFA profile revealed only minor changes. Propionate and acetate were both slightly reduced, which was against the general expectation that also in the hindgut the acetate to propionate ratio decreases with HC feeding [6,32]. The effect of decreasing acetate was also missing in other studies, where even increasing acetate was found with decreasing fecal pH [3,29], which suggests a reevaluation of the general statement. There is evidence that the hindgut mucosa preferentially metabolizes acetate rather than butyrate as the main energy source [6]. The decreasing pH could have impaired absorptive capacities or the energy metabolism of the epithelial cells. Butyrate increased with HC, which is the same trend as shown in other studies, investigating fecal or rumen samples [3,29,31]. Another possible explanation is that because the fecal pH was never measured below 7.0 during our whole experiment, the highest abundant family *Ruminococcaceae* and other acetate producers, which require a pH around 7.0 to produce acetate as end product [33], kept the acetate levels stable.

The microbial-fermentation end products that we measured in the feces, might have also been subjected to diurnal variation as it is known from the rumen [31] or other studies investigating feces [3]. There is first evidence from human and mice studies, that also the gut microbiome undergoes circadian changes [34]. In the current study, fecal samples were taken only at one time point before morning milking and feeding, where ruminal pH was actually highest [17]. This sampling procedure includes the risk that we probably have missed a diurnal fecal-pH drop and diurnal changes in SCFA after feeding. However, the strength of this sampling time point before morning feeding is that the effects that were detected must be detrimental because they occur even during a period when ruminal pH was recovered [17]. The increase in total SCFA during HC feeding proves excessive fermentation activity in the hindgut and indicates the potential for hindgut acidosis [6]. The significant decrease of bacterial diversity and richness, their relevant correlation with fecal pH and SCFA, and the clear separate clustering of MC to HC samples on distance metrics, proves the hypothesis of a dysbiotic microbial community due to bypass substrates [29]. In general, the alpha and beta diversity results give a similar picture as in the rumen when cows are switched from forage to 65% concentrate [31], proving that the feces are a valuable indicator for feed change analysis in terms of microbial community studies.

On bacterial family level, 71% changed in relative abundances with HC feeding. The highest abundant families found, do overlap with other studies investigating fecal microbiota in cattle [35] and sheep [36]. However, some studies, as [9], found almost 42% *Peptostreptococcaceae* in the rectum of cattle, followed by *Turicibacter*, and *Clostridium*, and [29] mainly *Planococaceae*, *Enterobacteriaceae*, *Moraxellaceae*, *Peptostreptococcaceae*, and *Erysipelotrichaceae*, which were all below 1% abundance or not present in our study. Mao et al. [9] found *Ruminococcaceae* with approximately 9.7% present in the rectal digesta. Mao et al. [29] detected, like in the current study, *Ruminococcacea* and *Lachnospiraceae* within their most abundant families. All mentioned studies used similar DNA-extraction protocols as in the current experiment, however different sets of primers. This could be a major contributing factor to the inhomogeneity of taxonomic assignment in cow-fecal samples and limits the possibility to compare studies according to their feeding regime [37]. Our highest abundant family *Ruminococcaceae* showed only a decrease by 1.05-fold relative abundance from the MC to the end of HC feeding. Although some *Ruminococcaceae* species are known to be rather acid-sensitive [38], they might have already adapted to the moderate level of carbohydrates and an increase by 20% was not enough to impair them further. Moreover, the fecal pH we measured had as minimum value 7.12 in HC, which is within the range of optimal pH for *Ruminococcaceae* and fiber degrading bacteria in general, as mentioned above [38]. The three low abundance families *Succinivibrionaceae*, *Fusobacteriaceae*, and *Campylobacteraceae* increased strongly with HC feeding (16.1-, 6.2-, 6.3-fold, respectively). These families are all Gram-negative representatives and include potential pathogenic species for which shedding could be stimulated by the high grain feeding because the bypass nutrients and lower pH provide a niche for those groups [39,40,41].

### 4.2. Rumen Acidosis and Hindgut Dysbiosis in Different Lactations

The ≥4 L cows had a higher mean, maximum, and minimum ruminal pH and spent less time below pH 6.0 than 2ndL or 3rdL cows. This indicates that the cows with a higher number of lactations were able to cope better with the HC feeding than cows with two or three lactations. This can be contributed to the higher number of previous confrontations with concentrate feed and therefore adaptive mechanisms [10]. On average, the ≥4 L cows did not have SARA according to the definition of 5–6 h per day below pH 6.0 [42]. In contrast, the 3rdL cows already reached the SARA threshold in the MC phase and kept on having the lowest mean, minimum, maximum pH, and highest number of minutes below pH 6.0 over the course of the whole experiment. This contradicts the general assumption that the higher the number of lactations, the more capable to deal with high starch are the cows. Taking the total DMI and the DMI/kg BW into account, we can conclude that the 2ndL cows consumed the same amount of DM according to their body weight, but 3rdL cows consumed more total DM, which led to a stronger pH drop, due to a higher total amount of concentrate [43]. Interestingly, 2ndL cows spent more time ruminating per kg DMI than 3rdL cows, which is a second major contributor why 2ndL cows did not drop as much in ruminal pH [4]. The level of total DMI and chewing indexes were the same for 3rdL and ≥4 L cows. However, the ≥4 L cows consumed less DM according to their BW and spent less total time eating. The size of the gastrointestinal (GI) tract correlates in general to the BW [32,44] so that it could be hypothesized that bigger cows can cope more easily with high concentrate due to their anatomical bigger GI tract. In deer, it was shown that the size of the reticulorumen increases not only with BW, but also with age itself [45]. For cattle, however, this topic was hardly ever investigated. The absorption capacity of the rumen wall is possibly more efficient in ≥4 L cows, because cows with more than three lactations have a thicker rumen epithelium then cows with three or less lactations [13]. Together with a bigger rumen, this provides them with potentially a higher number of papillae or larger rumen papillae, resulting in an increased absorptive surface [12]. That a higher absorptive capacity might be more efficient than higher chewing activity in the removal of protons was already discussed in [46].

From this data, we conclude that the rumen of the 3rdL cows is not as adapted as the rumen of ≥4 L cows, but they are already at the same production level (DMI, milk kg) as the ≥4 L cows. This made them the most susceptible to SARA. The rumen of the 2ndL cows is also not as adapted, but due to their lower total DMI and higher chewing activity, they had a less strong drop in ruminal pH.

The difference of ruminal pH in the lactation groups was not reflected by the fecal pH. This is against the assumption that the more severe SARA is, the stronger is the acidosis in the hindgut [6]. One explanation could be the lack of continuous fecal-pH measurements leading to a miss of acidotic pH values after feeding. During our sampling time point, the fecal pH of all cows might have recovered to the same level. Nevertheless, we were able to detect higher total SCFA in 3rdL cows, which reflects higher fermentation activity due to bypass substrates. Four phyla and four high-abundant families changed in relative abundance in association with lactation. We hypothesize that previous adaptation to HC diet results in a memory and facilitates the adaptation of certain bacterial groups to certain available substrates.

Certain bacterial families followed a lactation number dependent trend, as *Alcaligenaceae*, *Anaeroplasmataceae*, *Erysipelotrichaceae*, *Succcinivibrionaceae*, *Coriobacteriaceae*, and *Peptococcaceae* decreased with increasing lactation number. *Porphyromonadaceae*, *Clostridiacaee*, *Spirochaetaceae*, S247, and *Barnseliellaceae* increased with increasing lactation number. These bacterial families seem to be more independent of the effects of the feeding model, such as low ruminal pH and higher total SCFA in 3rdL cows. They could be indicator phylotypes for age-dependent changes in the microbiome and lesser susceptibility to SARA. The results of Zhang et al. [47] support our data for *Elusimicrobia*, *Clostridium, Succinivibrio,* and *Lachnospiraceae* changes with the number of lactations.

## 5. Conclusions

In conclusion, the feeding of 60% concentrate, containing 27.7% starch, caused increased hindgut fermentation with a decrease in fecal pH, bacterial diversity, and a shift in bacterial families, suggesting hindgut dysbiosis. Our findings underline the need for more continuous fecal pH measurements to describe diurnal changes and set possible pH thresholds for hindgut acidosis. Cows with ≥4 L did not develop SARA with 60% concentrate feeding, but fecal pH decreased to the same extent as in 2ndL and 3rdL cows. The cows in 3rdL were at a similar level of DMI, chewing activity, and milk production as ≥4 L cows, but had a lower BW and likely a smaller rumen and a lower absorptive capacity in the GI tract. This made them less capable of managing high-starch diets and led to severe ruminal pH drops and a higher fermentation rate in the hindgut. The 2ndL cows also developed SARA, but with a less severe pH drop, due to their lower total DMI and higher rumination and eating time. Several bacterial families changed in relative abundance with the number of lactations and display indicator phylotypes for age. This indicates possible adaptive mechanisms of the fecal microbiome in cows that experienced more lactations before.

## Figures and Tables

**Table 1:** Fermentation parameters in feces changing from moderate- to high-concentrate feeding

Parameter	Feeding Phase ^1^				SEM	*p*-Value Phase
	MC	HCwk2	HCwk3	HCwk4		
Fecal pH (mean)	7.78 ^a^	7.51 ^b^	7.45 ^b,c^	7.39 ^c^	0.038	<0.001
Total SCFA (mmol/L)	55.7 ^b^	88.9 ^a^	83.0 ^a^	86.0 ^a^	6.07	<0.001
Acetate (%)	75.5 ^a^	73.9 ^b^	74.8	74.4	0.77	0.08
Propionate (%)	14.3 ^a^	13.4 ^b^	13.8 ^a^	13.1 ^b^	0.29	0.01
Butyrate (%)	4.3 ^c^	5.3^a^	5.2 ^a^	4.8 ^b^	0.17	<0.001
Valerate (%)	1.14 ^a^	1.11 ^a,b^	1.01 ^b^	0.99 ^b^	0.05	0.05
Caproate (%)	0.16 ^b^	2.09	0.46 ^b^	2.49 ^a^	0.819	0.05
iso-Butyrate (%)	3.9	3.9	4.3	3.8	0.24	0.48
iso-Valerate (%)	0.84 ^a^	0.36	0.46 ^b^	0.38 ^b^	0.047	<0.001

^1^ 16 dairy cows were fed a 40% concentrate diet (18.8% starch, DM base) for 1wk (MC), followed by four weeks of 60% concentrate diet (27.7% starch, DM base; HCwk1–4). ^a,b,c^ Different superscript letters indicate significant difference (*p* ≤ 0.05) or difference by trend (0.05 < *p* ≤ 0.1, in parentheses) between the feeding phases. SEM, Standard error of the mean.

**Table 2:** Alpha-diversity-parameters of the cow-fecal microbiome when switched from moderate- to high-concentrate diet

Parameter	Feeding Phase ^1^			SEM	*p*-Value Phase
	MC	HCwk2	HCwk4		
Goods coverage	0.920 ^b^	0.931 ^a^	0.934 ^a^	0.0026	<0.001
Shannon	8.85 ^a^	8.24 ^b^	8.19 ^b^	0.092	<0.001
Simpson	0.992 ^a^	0.983 ^b^	0.984 ^b^	0.0017	<0.001
Chao1	2813 ^a^	2418 ^b^	2349 ^b^	83.2	<0.001
Singletons	798 ^a^	687 ^b^	656 ^b^	26.6	<0.001

^1^ 16 dairy cows were fed a 40% concentrate diet (18.8% starch, DM) for 1wk (MC), followed by four weeks of 60% concentrate diet (27.7% starch, DM; HCwk1–4). ^a,b^ Superscript letters indicate a significant difference (*p* ≤ 0.05) between the feeding phases. SEM = Standard error of the mean.

**Table 3:** DMI, milk yield, chewing activity, milk contents, ruminal pH, fecal pH, and fecal SCFA profile in cows during second (2ndL, *n* = 5), third (3rdL, *n* = 6), or fourth or higher lactation (≥4 L, *n* = 5) along the feeding model (MC, 40% concentrate, 18.8% starch, for one week; HCwk1–4, 60% concentrate, 27.7% starch, for four weeks, DM base).

Parameter	MC			HCwk1			HCwk2			HCwk3			HCwk4			SEM	*p*-Value Lact.
	2ndL	3rdL	≥4 L	2ndL	3rdL	≥4 L	2ndL	3rdL	≥4 L	2ndL	3rdL	≥4 L	2ndL	3rdL	≥4 L		
Performance and Chewing																	
Milk yield (kg/day)	29.9 ^(b)^	34.4 ^(a)^	34.5 ^(a)^	30.6 ^b^	35.3 ^a^	35.2 ^(a)^	31.7 ^(b)^	36.2 ^(a)^	36.6 ^(a)^	31.4 ^b^	36.9 ^a^	36.2 ^a^	31.0 ^b^	34.9 ^(a)^	36.6 ^a^	1.66	<0.001
DMI (kg/day)	20.7 ^b^	23.5 ^a^	22.1	21.3 ^b^	24.3 ^a^	24.8 ^a^	21.9 ^(b)^	24.4	25.0 ^(a)^	22.9 ^b^	25.6 ^a^	24.9	22.8 ^b^	26.0 ^a^	25.6 ^a^	0.98	<0.001
DMI/BW (kg/kg)	3.2 ^a^	3.1 ^a^	2.7 ^b^	3.3	3.2	3.1	3.4 ^(a)^	3.2	3.1 ^(b)^	3.5 ^a^	3.4 ^(a)^	3.1 ^b^	3.5 ^a^	3.5 ^a^	3.2 ^b^	0.15	<0.001
Milk yield/DMI (kg/kg)	1.5	1.5	1.6	1.5	1.5	1.5	1.5	1.5	1.5	1.4	1.5	1.5	1.4	1.3	1.5	0.09	n.s.
Rumination min/day	569	581	573	522	530	556	n.a.	n.a.	n.a.	n.a.	n.a.	n.a.	533	548	553	20.7	n.s.
Eating min/day	377 ^a^	399 ^a^	292 ^b^	333	353 ^(a)^	288 ^(b)^	n.a.	n.a.	n.a.	n.a.	n.a.	n.a.	332	332	298	38.7	0.05
Rumination min/kg DMI	27.9 ^(a)^	25.2 ^(b)^	28.1 ^(a)^	25.6 ^a^	22.5 ^b^	23.3	n.a.	n.a.	n.a.	n.a.	n.a.	n.a.	23.1	20.4	21.0	1.07	0.04
Eating min/kg DMI	18.4 ^a^	16.9	14.2^b^	16.8 ^a^	14.9	11.8 ^b^	n.a.	n.a.	n.a.	n.a.	n.a.	n.a.	14.3	12.2	11.3	1.68	0.02
Rumen																	
Mean pH	6.39	6.30	6.70	6.27	6.18	6.56	6.26	6.20	6.50	6.22	6.17	6.46	6.24	6.14	6.42	0.220	<0.001 ^1^
Minimum pH	6.00	5.94	6.33	5.78	5.72	6.08	5.74	5.68	6.03	5.72	5.61	6.02	5.82	5.65	5.98	0.211	<0.001 ^1^
Maximum pH	6.71 ^b^	6.49 ^b^	7.09 ^a^	6.75 ^b^	6.53 ^b^	7.05 ^a^	6.70	6.49 ^b^	6.98 ^a^	6.67	6.50 ^b^	6.94 ^a^	6.57	6.42 ^b^	6.87 ^a^	0.110	<0.001
minutes pH<6.0	204	454 ^a^	21 ^b^	380	640 ^a^	151 ^b^	370	598 ^a^	99 ^b^	438	636 ^a^	127 ^b^	480	703 ^a^	96 ^b^	137.7	<0.001
Milk content																	
Fat (%)	3.8	3.9	3.9	n.a.	n.a.	n.a.	3.9	3.6	3.5	5.1 ^(a)^	4.0 ^(b)^	3.9 ^(b)^	3.6	3.7	3.4	0.44	0.08
Protein (%)	3.4	3.4	3.2	n.a.	n.a.	n.a.	3.5	3.5	3.5	3.5	3.6	3.5	3.6	3.6	3.6	0.10	n.s.
Lactose (%)	4.9	4.8	4.8	n.a.	n.a.	n.a.	4.8	4.7	4.7	4.8	4.7	4.7	4.8	4.7	4.7	0.06	0.06
Milk urea nitrogen (mg/dL)	26.9 ^a^	26.4 ^a^	20.5 ^b^	n.a.	n.a.	n.a.	19.0	18.2	16.6	21.9	21.5	18.3	25.5	22.2	22.5	1.53	0.01
Urea/DMI (mg/dL/kg)	1.31 ^a^	1.14 ^a^	0.93 ^b^	n.a.	n.a.	n.a.	0.86 ^(a)^	0.76	0.66 ^(b)^	0.96 ^(a)^	0.87	0.73 ^(b)^	1.12 ^a^	0.87 ^b^	0.88 ^b^	0.072	<0.001
Somatic cell count (10^3^)	49	40	93	n.a.	n.a.	n.a.	67	52	73	71	59	204	50 ^b^	41 ^b^	438 ^a^	96.3	0.01
Feces																	
Mean pH	7.75	7.79	7.81	n.a.	n.a.	n.a.	7.48	7.54	7.50	7.47	7.41	7.48	7.38	7.39	7.40	0.062	n.s.
Total SCFA (mmol/L)	52.3	57.5	56.9	n.a.	n.a.	n.a.	89.7	93.4	82.6	78.3 ^b^	100.7 ^a^	66.5 ^b^	88.6	86.7	82.8	8.83	0.03
Acetate (%)	74.9	75.6	75.8	n.a.	n.a.	n.a.	73.8	72.5 ^b^	75.6 ^a^	75.3	74.4	74.8	74.5	75.4	73.2	1.11	0.05
Propionate (%)	14.5	14.2	14.1	n.a.	n.a.	n.a.	12.7 ^(b)^	13.7 ^(a)^	13.7 ^(a)^	14.0	13.5	13.9	12.4 ^b^	13.0	13.9 ^a^	0.40	0.02
Butyrate (%)	4.3	4.1	4.4	n.a.	n.a.	n.a.	5.3	5.2	5.3	5.5	5.1	5.0	5.0	4.8	4.7	0.25	n.s.
Valerate (%)	1.22	1.11	1.10	n.a.	n.a.	n.a.	1.3 ^a^	1.03 ^b^	0.99 ^b^	1.07	0.97	1.00	1.04	0.91	1.04	0.079	0.02
Caproate (%)	0.15	0.15	0.19	n.a.	n.a.	n.a.	2.32	3.57 ^(a)^	0.07 ^(b)^	0.07	1.11	0.08	3.14	1.95	2.49	1.329	0.10
iso-Butyrate (%)	3.97	3.85	3.80	n.a.	n.a.	n.a.	4.20	3.49	4.06	3.71 ^(b)^	4.27	4.87 ^(a)^	3.53	3.51	4.34	0.379	0.07
iso-Valerate (%)	0.94 ^a^	0.90 ^a^	0.66 ^b^	n.a.	n.a.	n.a.	0.41	0.37	0.28	0.43	0.51	0.42	0.38	0.31	0.46	0.071	0.01

^a,b,c^ Different superscript letters indicate significant difference (*p* ≤ 0.05) or difference by trend (0.05 < *p* < 0.1, in parentheses) between lactation groups within one feeding phase; n.s. not significant (*p* ≥ 0.1); ^1^ Mean and minimum pH were in each feeding phase higher in ≥4 L than in 2ndL and 3rdL cows. SEM, Standard error of the mean; n.a., not analyzed.

## Supplementary Materials

The following are available online at https://www.mdpi.com/2076-2615/10/10/1727/s1, Figure S1: Changes in relative abundance (%) of the fecal microbiome on family level; Table S1: Chewing parameters measured with rumination halters in cows during second (2ndL, *n* = 5), third (3rdL, *n* = 6), or fourth or higher lactation (≥4 L, *n* = 5) along the feeding model (MC, 40% concentrate, 18.8% starch, for one week; HCwk1–4, 60% concentrate, 27.7% starch, for four weeks, DM base).
