# Supplementary material for: Starch-Rich Diet Induced Rumen Acidosis and Hindgut Dysbiosis in Dairy Cows of Different Lactations
Source: Animals (Basel). 2020 Sep 23;10(10):1727. doi: 10.3390/ani10101727 (PMC7598178; doi:10.3390/ani10101727)
Supplement: Supplementary file 1 [file animals-10-01727-s001.zip › animals_supplementaryfiles/Supplemental Figure S1_Neubauer et al.pdf]

Family	Total rel. ab. %	Feeding phase MC	HCwk2	HCwk4	SEM	*P* - value MC-HCwk2	MC-HCwk4	HCwk2-HCwk4	Phase
Ruminococcaceae	61.9%	64.12	61.76	60.64	1.342	n.s.	0.027	n.s.	0.075
Lachnospiraceae	6.18%	4.04	6.55	7.53	0.426	<0.001	<0.001	n.s.	<0.001
Bacteroidaceae	4.54%	5.17	4.33	4.01	0.453	0.019	0.002	n.s.	0.005
Rikenellaceae	3.59%	4.67	3.44	2.94	0.228	<0.001	<0.001	0.093	<0.001
RF16	2.75%	2.47	3.10	2.65	0.248	0.075	n.s.	n.s.	n.s.
Porphyromonadaceae	2.33%	1.73	2.32	3.03	0.292	n.s.	0.001	0.059	0.004
Paraprevotellaceae	1.95%	2.30	1.91	1.69	0.159	0.037	0.001	n.s.	0.004
Clostridiaceae	0.96%	0.74	0.72	1.36	0.361	n.s.	n.s.	n.s.	n.s.
Prevotellaceae	0.87%	0.39	1.00	1.12	0.145	0.003	<0.001	n.s.	0.001
Spirochaetaceae	0.47%	0.437	0.534	0.417	0.0553	n.s.	n.s.	0.079	n.s.
p253418B5	0.45%	0.528	0.369	0.504	0.0891	n.s.	n.s.	n.s.	n.s.
Victivallaceae	0.39%	0.967	0.149	0.099	0.0935	<0.001	<0.001	n.s.	<0.001
Alcaligenaceae	0.36%	0.268	0.557	0.280	0.0584	<0.001	n.s.	<0.001	<0.001
S247	0.31%	0.211	0.298	0.401	0.0376	n.s.	0.001	0.054	0.003
Anaeroplasmataceae	0.30%	0.224	0.369	0.332	0.0493	0.047	n.s.	n.s.	n.s.
Erysipelotrichaceae	0.24%	0.233	0.235	0.250	0.0199	n.s.	n.s.	n.s.	n.s.
Mogibacteriaceae	0.23%	0.295	0.223	0.216	0.0303	0.007	0.002	n.s.	0.004
Fibrobacteraceae	0.21%	0.242	0.190	0.189	0.0536	n.s.	n.s.	n.s.	n.s.
Christensenellaceae	0.13%	0.164	0.112	0.122	0.0158	0.022	0.051	n.s.	0.047
Succinivibrionaceae	0.09%	0.008	0.125	0.130	0.0383	0.024	0.014	n.s.	0.024
Coriobacteriaceae	0.08%	0.029	0.033	0.156	0.0280	n.s.	0.002	0.004	0.003
Barnesiellaceae	0.08%	0.106	0.056	0.059	0.0111	0.003	0.004	n.s.	0.003
Veillonellaceae	0.03%	0.033	0.039	0.031	0.0052	n.s.	n.s.	n.s.	n.s.
Peptostreptococcaceae	0.03%	0.030	0.024	0.026	0.0126	n.s.	n.s.	n.s.	n.s.
Elusimicrobiaceae	0.02%	0.031	0.018	0.022	0.0085	n.s.	n.s.	n.s.	n.s.
BS11	0.02%	0.018	0.014	0.022	0.0028	n.s.	n.s.	0.037	n.s.
Sphaerochaetaceae	0.01%	0.014	0.012	0.013	0.0029	n.s.	n.s.	n.s.	n.s.
Odoribacteraceae	0.01%	0.020	0.010	0.008	0.0028	0.013	0.003	n.s.	0.007
F16	0.01%	0.023	0.007	0.005	0.0019	<0.001	<0.001	n.s.	<0.001
R445B	0.01%	0.020	0.003	0.001	0.0044	0.006	0.002	n.s.	0.003
Fusobacteriaceae	0.004%	0.001	0.004	0.008	0.0023	n.s.	0.002	0.074	0.008
Peptococcaceae	0.004%	0.005	0.005	0.004	0.0013	n.s.	n.s.	n.s.	n.s.
Acetobacteraceae	0.004%	0.006	0.002	0.006	0.0011	0.026	n.s.	0.021	0.035
Campylobacteraceae	0.004%	0.001	0.008	0.004	0.0034	n.s.	n.s.	n.s.	n.s.
Desulfovibrionaceae	0.004%	0.006	0.003	0.002	0.0017	n.s.	n.s.	n.s.	n.s.
Caulobacteraceae	0.003%	0.003	0.002	0.005	0.0013	n.s.	n.s.	0.099	n.s.
Verrucomicrobiaceae	0.003%	0.005	0.001	0.002	0.0014	0.009	0.017	n.s.	0.016
Turicibacteraceae	0.002%	0.003	0.001	0.001	0.0016	0.011	0.040	n.s.	0.025
Unassigned family	11.44%	10.47	11.51	11.72	0.673	0.056	0.019	n.s.	0.043
Relative abundance	high								low

Supplemental Figure S1. Changes in relative abundance (%) of the fecal microbiome on family level. A group of 16 dairy cows where switched from moderate concentrate (MC, 40% concentrate, 18.8% starch, DM, 1 wk) to high-concentrate (HC, 60% concentrate, 27.7% starch, DM) diet for four weeks (HCwk1-4).

SEM standard error of the mean; n.s. not significant ($P \geq 0.1$).
