# Supplementary material for: Starch-Rich Diet Induced Rumen Acidosis and Hindgut Dysbiosis in Dairy Cows of Different Lactations
Source: Animals (Basel). 2020 Sep 23;10(10):1727. doi: 10.3390/ani10101727 (PMC7598178; doi:10.3390/ani10101727)
Supplement: Supplementary file 1 [file animals-10-01727-s001.zip › animals_supplementaryfiles/Supplemental Table_S1_Neubauer et al..pdf]

Supplemental Table S1. Chewing parameters measured with rumination halters in cows during second (2ndL, n = 5), third (3rdL, n = 6), or fourth or higher lactation (≥4L, n = 5) along the feeding model (MC, 40% concentrate, 18.8% starch, for one week; HCwk1-4, 60% concentrate, 27.7% starch, for four weeks, DM base).

Parameter	MC			HCwk1			HCwk4			SEM	p-value Lact.
	2ndL	3rdL	≥4L	2ndL	3rdL	≥4L	2ndL	3rdL	≥4L		
Chewing activity											
Total chewing min/d	947	987[a]	864[b]	857	888	845	866	884	849	48.3	0.06
Eating chews n/d	27941[a]	29162[a]	18748[b]	24131	25822	19853	23583	22963	19456	3787.0	0.08
Total chews n/d	65163[a]	66435[a]	53435[b]	57241	59049	53501	56574	57003	52371	4565.3	0.07
Rumination boli n/d	565[b]	651[a]	587	506[(b)]	576[(a)]	552	523	587	557	32.9	0.08
Chews/Bolus	65.3[a]	56.8[b]	58.2[b]	64.4[a]	56.3[b]	60.5	62.7	57.6	58.8	2.16	0.03
Total chews min/kg DMI	46.3	42.3	42.2	42.5[a]	37.5[b]	35.0[b]	37.5[(a)]	32.8[(b)]	32.3[(b)]	2.16	0.01
Rumination chews n/g DMI	1.8[a]	1.6[b]	1.7	1.6[a]	1.4[b]	1.4[b]	1.4	1.3	1.3	0.06	<0.01
Eating chews n/kg DMI	1.4[a]	1.2	0.9[b]	1.2[a]	1.1	0.8[b]	1.0	0.9	0.7	0.16	0.02
Total chews n/kg DMI	3.2[a]	2.8	2.6[b]	2.8[a]	2.5[b]	2.2[b]	2.5[a]	2.1	2.0[b]	0.19	<0.01

[a,b,c] Different superscript letters indicate significant difference ($p \leq 0.05$) or difference by trend ($0.05 < p < 0.1$, in parentheses) between lactation groups within one feeding phase.

SEM Standard error of the mean
